# Diagnostic and Therapeutic Approach to Thoracic Outlet Syndrome

**DOI:** 10.3390/tomography10090103

**Published:** 2024-09-01

**Authors:** Stefania Rizzo, Cammillo Talei Franzesi, Andrea Cara, Enrico Mario Cassina, Lidia Libretti, Emanuele Pirondini, Federico Raveglia, Antonio Tuoro, Sara Vaquer, Sara Degiovanni, Erica Michela Cavalli, Andrea Marchesi, Alberto Froio, Francesco Petrella

**Affiliations:** 1 Imaging Institute of Italian Switzerland (IIMSI), Ente Ospedaliero Cantonale, via Tesserete 46, 6900 Lugano, Switzerland; stefania.rizzo@eoc.ch; 2 Facoltà di Scienze Biomediche, Università della Svizzera Italiana, via G.Buffi 13, 6900 Lugano, Switzerland; 3 Division of Radiology, Fondazione IRCCS San Gerardo dei Tintori, 20900 Monza, Italy; cammilloroberto.taleifranzesi@irccs-sangerardo.it; 4 Division of Thoracic Surgery, Fondazione IRCCS San Gerardo dei Tintori, 20900 Monza, Italy; andrea.cara@irccs-sangerardo.it (A.C.); enricomario.cassina@irccs-sangerardo.it (E.M.C.); lidia.libretti@irccs-sangerardo.it (L.L.); emanuele.pirondini@irccs-sangerardo.it (E.P.); federico.raveglia@irccs-sangerardo.it (F.R.); antonio.tuoro@irccs-sangerardo.it (A.T.); sara.vaquer@unimi.it (S.V.); sara.degiovanni@unimi.it (S.D.); 5 Division of Plastic and Reconstructive Surgery, Fondazione IRCCS San Gerardo dei Tintori, 20900 Monza, Italy; ericamichela.cavalli@irccs-sangerardo.it (E.M.C.); andrea.marchesi@irccs-sangerardo.it (A.M.); 6 Division of Vascular Surgery, Fondazione IRCCS San Gerardo dei Tintori, 20900 Monza, Italy; alberto.froio@unimib.it; 7 Department of Medicine and Surgery, University of Milano-Bicocca, 20900 Monza, Italy

**Keywords:** thoracic outlet syndrome, CT, MRI, US, diagnosis, treatment, surgery

## Abstract

Thoracic outlet syndrome (TOS) is a group of symptoms caused by the compression of neurovascular structures of the superior thoracic outlet. The knowledge of its clinical presentation with specific symptoms, as well as proper imaging examinations, ranging from plain radiographs to ultrasound, computed tomography and magnetic resonance imaging, may help achieve a precise diagnosis. Once TOS is recognized, proper treatment may comprise a conservative or a surgical approach.

## 1. Introduction

Thoracic outlet syndrome (TOS) is a group of symptoms caused by the compression of neurovascular structures of the superior thoracic outlet [1]. The thoracic outlet presents three anatomic districts through which the subclavian vein, subclavian artery and brachial plexus structures pass to reach the arms: the interscalene triangle, the costoclavicular space, and the retropectoralis minor space [2].

Both neurogenic and vascular components may contribute to TOS symptoms: compression of the subclavian vein and artery causes edema, swelling, cyanosis, and decreased blood flow to the arms; on the other hand, pain, dysesthesia, numbness, and weakness are usually due to neurogenic involvement [3].

Although clinically classified into venous, arterial and neurogenic, TOS was divided by Wilbourn in 2001 into 5 groups: (1) arterial vascular, (2) venous vascular, (3) traumatic neurovascular or simply traumatic, (4) neurogenic, and (5) non-specific or mixed [4].

Neurogenic TOS accounts for 80% to 90% of all diagnosed cases of TOS, with an incidence of 1 per million people [5]. Cases of vascular TOS are less frequent; in fact, venous TOS accounts for 3% to 4% and the arterial form represents about 1% to 2% of all diagnosed cases of TOS [6].

Clinical evaluation still plays a pivotal role in TOS diagnosis, combining a proper history and physical examination with provocative tests. However, the diagnosis of TOS is often a diagnosis of exclusion, and its confirmation can be challenging, lacking specificity.

Imaging studies and electrodiagnostic tests may complete the diagnostic work-up, confirming neurovascular compression and disclosing the location and extent of the compression. There is no standard codification of therapeutic options, the surgical approach being the standard option in cases of vascular TOS, whereas a conservative approach and physiotherapeutic treatment is recommended as the first option in neurogenic forms, hence the need for a reliable and clear imaging-based diagnosis [7].

This review offers a broad up-to-date overview of clinical presentation, diagnostic and therapeutic (non-surgical and surgical) approaches to TOS.

## 2. Clinical Presentation and Clinical Evaluation

Early recognition of signs and symptoms of TOS, especially for neurological TOS, is an important step in preventing disease progression and in planning the right treatment at the right time. Patients presenting with neurological TOS often present with a previous history of neck trauma, such as after car accidents, or related to repetitive work stress [8].

Biomechanical factors may be helpful as adjunctive diagnostic tools, particularly those of the shoulder joint and pelvis. Indeed, muscle imbalance at the shoulder level, such as in the presence of hypertrophy of the pectoralis minor or hypertrophy of the scalene and sternocleidomastoid muscles, can directly cause an entrapment of the brachial plexus, as well as of the subclavian artery and/or of the subclavian vein. In addition, pelvic alignment should not be disregarded, as it may affect the posture of the upper body, thus indirectly contributing to the function of the thoracic outlet [9]. Evaluating and treating overall posture and addressing muscle imbalances may help minimize entrapment and give the patient initial relief from the TOS.

Physical inspection of the patient is the first step of the diagnostic algorithm of suspected TOS. In cases of monolateral disease, the affected limb should be carefully compared to the contralateral non affected arm. In addition, the cervical spine and neck—including the scalene triangle—should be properly inspected and an examination of the shoulder should routinely be performed. A full neurological examination of the upper limbs, a peripheral vascular examination and the performance of provocative maneuvers complete the physical inspection. Swelling and cyanosis of the fingers and hands might suggest venous TOS; on the other hand, Raynaud’s phenomenon, upper limb ischemia, finger ulceration and peripheral embolization can support the diagnosis of arterial TOS. In neurogenic TOS, the hands might show muscular atrophy signs, although not frequently reported; moreover, palpation at sites of compression—such as the supraclavicular scalene triangle or subcoracoid pectoralis minor insertion site—can provoke pains and other symptoms related to neurological TOS [10].

The most commonly used physical provocative tests are:

Allen test: the patient is asked to elevate their hand and to clench their fists for around 30 s. Then pressure is applied—by the examinator—over the ulnar and the radial arteries so as to occlude both of them. With the arm still up, the patient is asked to release the fingers: the hand should appear pale. After releasing the pressure over the ulnar artery and maintaining it over the radial artery, the color of the skin should return normal in a few seconds; in this case, the Allen test is defined as negative. If the skin remains pale, the Allen test is considered positive, thus suggesting an insufficient ulnar artery supply.

Wright test: facing the head of the patient forward, the arm to be examined is passively abducted and externally rotated to 90° without tilting the head. The elbow is flexed no more than 45 degrees. The arm is then held for 1 min while the examiner monitors the radial pulse and patient symptoms. Then the test is repeated, having the patient hold the extremity at the end range of abduction. The test is defined positive for thoracic outlet syndrome if a decrease in the radial pulse is observed or reproduction of the patient’s symptoms is provoked.

Adson test: The arm to be examined of the seated or standing patient is abducted 30° at the shoulder and maximally extended. The radial pulse is monitored by the examiner, who grasps the patient’s wrist. The patient is then asked to extend the neck and turn the head toward the symptomatic side, taking a deep breath and holding it. The examiner then compares the radial pulse in this position. The test is defined as positive when a marked decrease or disappearance of the radial pulse is observed. A comparative test on the non-affected side is recommended.

Roos test: the patient has both arms in the 90° abduction–external rotation position with shoulders and elbows in the frontal plane of the chest. The patient is asked to open and close both hands slowly over a 3 min period. Usually only limited forearm muscle fatigue and minimal distress are reported; the test is defined as positive when paresthesia in the forearm and fingers as well as pain in the neck and shoulder are reported. In cases of venous TOS, swelling and cyanosis might appear; in cases of arterial TOS, pallor of the elevated hands is observed and reactive hyperemia might be reported when the arms are lowered.

## 3. Patho-Physiology

Patho-physiology of TOS may account for its clinical presentation and symptoms. Indeed, venous TOS, previously considered to be the result of an acute thrombosis of the subclavian vein, is now better understood as a chronic disease process due to repetitive venous injury during arm use and elevation in the setting of normal anatomic arrangements in the costoclavicular space. The chronicity of this state is reflected as a cycle of injury and tissue repair, with progressive deposition of perivenous constricting scar tissue, which may remain asymptomatic for many years as the body is able to develop collateral draining pathways until an acute thrombus in the main subclavian vein develops [11].

Subclavian artery stenosis related to TOS is usually related to a prolonged and sustained compression of the subclavian artery, as seen in the setting of bony abnormalities. The poststenotic dilatation of the artery leads to aneurysmal wall degeneration, with intimal ulceration and thrombus formation that is prone to distal embolization [11].

On the other hand, in neurogenic TOS pathophysiology, whose symptoms are related to brachial plexus compression, recently, a major role has been attributed to the dynamic role of the pectoralis minor muscle. Indeed, the hyperactivity of this muscle results in shortening and fibrosis, which leads the scapula to assume a chronically protracted posture, thus decreasing the volume of the retropectoralis minor space [12].

As long as the pathophysiology of TOS syndrome is better known we may expect that improved diagnostic and therapeutic options might become available. For instance, there has been growing recent literature about the opportunistic use of imaging examinations to quantify the body composition through CT and MRI [13], as well as about other minable quantitative metrics from MRI [14], but currently no studies have yet evaluated if these tools may improve the diagnostic and therapeutic options of TOS. Furthermore, there is still the need for studies evaluating the long-term outcomes of therapeutic approaches according to dedicated measures of quality of life [15].

## 4. Imaging Techniques

Nowadays, many different imaging techniques are available in order to rule in or rule out the diagnosis of TOS, but one can be more appropriate than another according to the specific type of TOS. Indeed, the ACR appropriateness criteria clearly indicate dedicated diagnostic workups for arterial, venous and neurologic TOS [16]. More specifically, for neurogenic TOS, MRI of the chest (without and with contrast medium) and chest radiography are considered usually appropriate; for venous TOS, catheter venography, Doppler US, chest CT with contrast medium and chest radiography are considered usually appropriate; for arterial TOS, CT with contrast medium, MR angiography with and without contrast medium, chest radiography, Doppler US and arteriography are usually considered appropriate.

In the following paragraphs, each imaging technique is explained in more detail.

### 4.1. Standard Radiograph

Standard radiographs of the chest and of the cervical spine are frequently used as an initial imaging modality as they can show anatomical abnormalities, such as supranumerary cervical ribs, clavicular and vertebral bone anomalies (e.g., a prominent C7 transverse process), as well as intervertebral discopathies causing cervico-brachial neuralgia. Furthermore, a plain chest radiograph may rule out the presence of large tumors within the thoracic cavity [8,17], although its negative predictive value is low for small tumors.

### 4.2. Ultrasound (US)

US is an excellent technique for evaluating arterial or venous pathology throughout the body. Compared with other imaging modalities, US is relatively low cost, requires no ionizing radiation, is often available at the bedside, and it is noninvasive. Therefore, as for other vascular pathologies, after an accurate anamnesis that leads to a clinical suspect, an US examination may help to tailor the diagnosis, often with the adjunct of a duplex scan investigation [18]. Doppler ultrasound is a noninvasive adjunctive component of this imaging examination that can easily be performed during dynamic maneuvers. The technique involves B-mode US and Doppler study of the subclavian vessels and is typically performed at rest (neutral position) and with provocative maneuvers [16], that may trigger flow acceleration followed by turbulence and an arrest in signal propagation [19,20]. Although the main advantage of US is the ability to directly compare the induced symptoms and concurrent direct vessel visualization, there is debate in the literature as to the significance of the imaging findings, particularly with respect to maneuvers to minimize the thoracic outlet and associated spaces [19].

According to the 2020 American College of Radiology (ACR) Appropriateness Criteria for imaging in the diagnosis of TOS, US is a cost-effective tool, especially for patients presenting with symptoms related to arterial or venous compression [16]. However, the sonographic diagnosis of compressive effects upon the brachial plexus may be challenging and may miss regional pathology, such as a pancoast tumor or cervical spondylopathy [16].

One of the major benefits of US is the possibility of performing dynamic provocation maneuvers and recording their hemodynamic effects on subclavian vessels, as well as the lack of radiation exposure, which is particularly relevant when dealing with young patients, as in the usual clinical setting of TOS. Stegemann et coll. recently tried to assess whether US could work as a feasible alternative to digital subtraction angiography (DSA) for diagnosing TOS [21]. They concluded that US is extremely reliable in the diagnostic process of arterial TOS, even more than DSA; their study, in fact, disclosed high US inter-rater agreement when performed according to a standard protocol, thus advocating US as the gold standard approach for the diagnosis of hemodynamically relevant compression of arteries in arterial TOS [21]. However, other authors reported that provocative maneuvers for dynamic ultrasound evaluation could result in painful reactions, thus limiting their diagnostic accuracy [22].

US may also facilitate the diagnosis of a neurological TOS. Indeed, in a prospective study dedicated to 20 patients with TOS, a novel and distinctive ultrasonographic sign (the “wedge-sickle sign”) was identified in 19/20 patients [23]. This sign refers to a hyper-echoic fibromuscular structure at the medial edge of the middle scalene muscle that causes enlargement and hypo-echogenicity of the lower trunk of the brachial plexus.

Contrast enhanced US (CEUS) is a developing modality that supersedes standard vascular ultrasound imaging and complements other modalities in the evaluation of vascularized lesions and vessels [24,25,26]. Administered intravenously, the US contrast consists of microbubbles filled with gas, surrounded by a stabilizing shell. This type of imaging may help to quantify vascular pathologies by acting as an intravascular tracer of ultrasound energy. Based on these properties, CEUS has the potential to play a role in the management of vascular pathologies, although its role in TOS is unclear [26].

The role of US may be limited in the evaluation of neural structures. Indeed, it can be difficult to properly visualize the nerves, as they are very thin structures that may be obscured by shadows of the bony structures.

Moreover, the well-known limitation of US is the dependency on operator experience.

US can also be used for guidance in injecting botulinum toxin into the anterior scalene muscles to foresee the clinical response to surgical decompression. According to Torriani et coll., patients disclosing complete resolution or reduction of their symptoms after botulinum toxin injection are more likely to benefit from surgical treatment of TOS by first rib resection or scalene release [27].

### 4.3. Computed Tomography (CT)

Suspected TOS should always be clearly indicated in the CT imaging request because the acquisition protocol should be adapted to this indication.

Imaging of the thoracic outlet often requires specific protocols and positioning that is not inherent in standard CT or magnetic resonance imaging (MRI) neck, cervical spine, or chest protocols. CT evaluation of TOS is typically performed in “neutral” and “stressed” positions. Images are obtained from the elbow to the aortic arch with the arms adducted (neutral), followed by abduction (stressed) and repeat imaging. When contrast is indicated, scan acquisition is typically performed with a contralateral antecubital intravenous (IV) injection, with either an empiric scan delay of 15 to 20 s or bolus tracking over the ascending aorta [27,28]. Some centers add the additional step of placing the contralateral arm in abduction (with the symptomatic ipsilateral arm in the neutral position) in order to minimize streak artifact.

Raptis et al. [29] suggest performing CT as the imaging technique of choice in the diagnosis of TOS when MR angiography is not feasible due to patient conditions (such as claustrophobia), or MR incompatibility with implanted devices.

Raptis et al. suggest a CT protocol including an acquisition before the injection of contrast medium, followed by an arterial phase with half contrast medium bolus with the symptomatic arm adducted and the asymptomatic or less symptomatic arm abducted, and a second arterial phase after the injection of the remaining half contrast medium with the symptomatic arm abducted and the contralateral arm adducted [16,27]. However, Khalilzadeh et coll. suggest performing the standard examination with the patient’s arms in the neutral position without any additional scans after provocative maneuvers in order to reduce the radiation dose [3]. As a post-processing technique, they suggest reconstructing the CT images at both 1 mm and 2 mm slice thicknesses, with specific 3-dimensional views of the thoracic outlet [3]. Whenever venous TOS is suspected, an additional contrast medium infusion in the antecubital vein of the unaffected or less affected side (to minimize streak artifact due to contrast media in the injected vein) may be added to maximize the flow of the diluted contrast into the venous system of the affected arm. This might help to ameliorate the identification of any external compression, venous thrombus or any other anomaly potentially resulting in a clinically symptomatic venous TOS [3]. Jardin et coll. demonstrated the advantages of CT angiography performed during rest and compression maneuvers for the study of thoracic outlet vascular structures. This technique allows excellent visualization of any vascular stenosis due to both soft tissue and bone anomalies; moreover, it allows easy detection of any predisposing anatomic factors, thus permitting an effective differential diagnosis with other pathologic conditions like apical lung tumors [30]. Finally, according to the ACR criteria, all of the following elements are essential: timing of acquisition according to contrast medium administration and reconstruction/reformatting of 3-D renderings for arterial evaluation rather than for venous evaluation [16].

### 4.4. Magnetic Resonance Imaging

MRI imaging represents an optimal noninvasive technique in patients with suspected neurological TOS [16,29]. The MRI protocol should be performed by high resolution T1- and T2-weighted sequences on sagittal and axial planes to evaluate the anatomic spaces both with the symptomatic arm adducted and abducted, completed with contrast-enhanced arterial and venous 3D sequences [16]. Visualization of the brachial plexus and cervical spine and dynamic evaluation of neurovascular bundles in the cervico-thoraco-brachial region may be of great help in supporting a clinical diagnosis. Additional MRI findings may be edema and loss of fat surrounding the brachial plexus with arm abduction.

**Figure 1:**
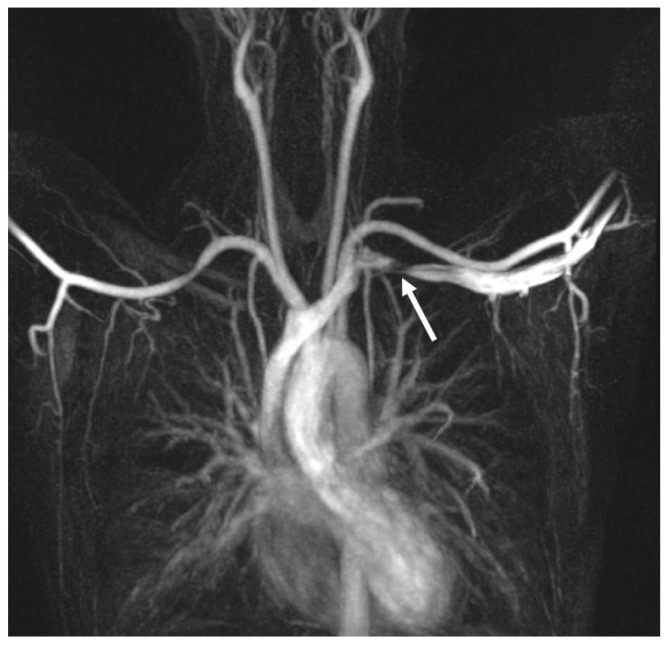
MRI disclosing left subclavian vein stenosis (white arrow) after ipsilateral arm abduction.

For suspected vascular TOS, when the administration of contrast medium is not advised, it is possible to use a modified MRI protocol with time-of-flight non-contrast-enhanced imaging [29]. Khalilzadeh et coll. suggest the use of a 3-Tesla MRI scanner for studying TOS with phased-array body and neck coils [3]. The first part of the exam is focused on the affected side and the second one on the non-affected side. Their protocol consists of localizing sequence, followed by coronal, sagittal and axial T1-weighted high-resolution images, and sagittal/coronal T2-weighted images in order to correctly evaluate the anatomy, cervical radiculopathy, the brachial plexus muscular attachment and site of compression [29]. The use of post-contrast, gadolinium-enhanced imaging is limited to ruling out or confirming the clinical suspicion of neoplastic tissue or infections as a source of the TOS symptoms. During the first part of the examination, the patient is in the supine position and both arms are at their sides with both palms facing up. The second part of the exam can be performed with one arm in an abduction external rotation position, with the head turned toward the side being examined [3] (Figure 1). Indeed, it has been demonstrated that changing the arm positioning can change the diameter of the subclavian vein because the anatomical relationship of the subclavian vein with its adjacent anatomical structures will change accordingly [31].

## 5. Neuro-Electric Tests

Neurogenic TOS is a very rare clinical condition with an estimated prevalence of 1 per 1,000,000 persons [32]. It is due to a chronic compression of C8–T1 nerve roots with or without lower trunk brachial plexopathy caused by compressive action of a supernumerary cervical rib or a fibrous band that arises from an extended C7 transverse process [33]. Because median innervated hand intrinsic muscles usually receive T1 dominant innervation and other hand intrinsic muscles receive C8 dominant- or C8/T1 innervation [32], in this clinical setting there is the typical syndrome defined as Gilliatt–Sumner hand. This syndrome is characterized by a group of symptoms consisting mainly of atrophy of the abductor pollicis brevis muscle, further atrophy of the interosseous and abductor digiti minimi muscles, and normal sensation in the region that receives innervation by the median nerve, including the thenar eminence [34]. Electrodiagnostic study plays a pivotal role in documenting neurogenic TOS: a median-motor, ulnar and medial antebrachial cutaneous sensory anomalous pattern due to T1 prominent involvement is typically reported; electrodiagnostic study also helps discriminate neurogenic TOS from other mimicking diseases [35]. When in doubt of osteomuscular anomalies potentially resulting in neurogenic TOS, standard chest radiographs are taken to rule out or prove the presence of a supernumerary cervical rib or extended C7 transverse process. According to Kim et coll., neurogenic TOS can be defined as T1 predominant lower roots/trunk brachial plexopathy characterized by abductor pollicis brevis muscle prevalent motor weakness, thenar area prevalent muscular atrophy, high sensitivity of medial antebrachial cutaneous nerve conduction study and a high prevalence of abnormalities in the abductor pollicis brevis with needle electromyography [36].

## 6. Therapeutic Options

### 6.1. Non-Surgical Approach

Physical therapy should be the first therapeutic option for TOS: in fact, it might be effective at decreasing symptoms, thus allowing return to work and improving arm function. It relies on patient education, pain control, range of motion, nerve gliding techniques, strengthening and stretching [37].

The aim of the initial stage is to decrease the patient’s symptoms. Once the patient has control over their symptoms, therapy should focus on addressing the tissues that create structural limitations of motion and compression. Massage, strengthening of the elevator scapulae, sternocleidomastoid and upper trapezius, stretching of the pectoralis, lower trapezius and scalene muscles, postural correction and relaxation of shortened muscles are the cornerstone of physical therapy at this stage. Manipulative treatment to mobilize the first rib is not usually recommended; whenever indicated, it should be carefully carried out and only after a thorough assessment as it might cause irritation and pain symptoms [38]. After surgical treatment of TOS, physical therapy should be immediately started to prevent scar tissue and return the patient to full function.

Thrombolysis is a non-surgical approach that might be effective in venous TOS. There are two different techniques that can be used in this setting: catheter-directed infusion of a thrombolytic agent or pharmacomechanical thrombolysis. Catheter-directed infusion consists in allocating a fenestrated catheter into the clot and delivering a thrombolytic agent through it for a variable period of time, depending on the extent and age of the clot. For a successful result the thrombus must be soft and not too old, so as to be easily traversed by the catheter; an old thrombus not amenable to a safe catheter crossing is almost considered a contraindication to this maneuver. Tissue plasminogen activator (tPA) is the most commonly used drug in this setting after assessing that the patient does not present any major contraindication to its use, such as recent major thoracic or abdominal surgery as well as proven intracranial processes like neoplasms or stroke. The major benefits of this technique are its simplicity and rapidity; on the contrary, one the of the most important limitations is its reduced efficacy in the case of long-lasting thrombus. Although anecdotal results have been reported in some cases of thrombi treated after several months, the success rate decreases significantly when the thrombus is more than two weeks old [39].

Pharmacomechanical thrombolysis consists of using a mechanical thrombolytic device while simultaneously delivering a thrombolytic agent. Due to the double action performed by this technique, the main advantage of this procedure is the speed of clot resolution. In fact, the mechanical action of the device makes it possible to deliver the thrombolytic drug over a more extended area and to better fragment the thrombus. There are several pharmacomechanical thrombolytic devices that work in different ways: there is an impeller generating a vortex that aspirates clots into the impeller itself and fragments clots into micron sized parts; there is a motor-driven rotating basket providing more effective clot fragmentation, particularly in the case of long-lasting thrombi, although due to its potential contact with vessel walls, it could potentially result in endothelial damage; there are several devices working on the sole principle of lysed clot aspiration, although cases of brady-arrhythmias or heart block have sometimes been observed when applied too close to heart chambers [39]. Other devices work by infusing a thrombolytic agent between two endovascular balloons (one distal and the other proximal to the thrombus), resulting in trapping the lytic drug in that specific vessel segment. Another recently described device infuses the thrombolytic agent and creates ultrasound along the entire length of the device: this effect produces an increase in the thrombolytic effect by augmenting the spreading of the lytic drug into the thrombus. As there are randomized controlled trials comparing the previously described devices, current usage is usually determined by the comfort level and personal experience of the operator [39].

### 6.2. Surgical Approaches

#### 6.2.1. Transaxillary Approach

The transaxillary approach is often considered one of the best approaches in cases that do not require vascular reconstruction. First described by Roos et al. in 1966 [40], this approach allows good exposure of the anterior part of the first rib, subclavius muscle insertion and costoclavicular ligament. The patient lies in full lateral decubitus position (90°), with the affected side upward, exposing the axilla. An incision is made relatively low in the axilla to avoid entering the lymph nodal tissue; the latissimus dorsi medially and the pectoralis major anteriorly define the edge of the exposure. Care must be taken to preserve the long thoracic nerve posteriorly, as its injury might result in winged scapula; on the contrary, the intercostobrachial cutaneous nerve, emerging between the first and second ribs, can sometimes be preserved but often must be sacrificed for further exposure. It is paramount to correctly identify the first rib by properly visualizing the insertion of the anterior scalene muscles to prevent the inadvertent removal of the second rib, representing a serious technical mistake. At this point of the procedure, the lateral and the medial border of the first rib are cleared by using a combination of sharp and blunt dissection. Many authors suggest an almost complete removal of the first rib, avoiding sub periosteal partial resection that might result in regrowth and symptom recurrence. It is important to free the subclavian vein from any fibrotic ring and cicatricial tissue surrounding the vessel to properly expose the native and healthy venous adventitia. The costoclavicular ligament needs to be sectioned, as well as the origin of the subclavius muscle from the junction of the 1st rib and its costal cartilage, to maximize vein exposure. Possible complications of this approach are pneumothorax, chylothorax—in particular on the left side—and winged scapula in the case of long thoracic nerve damage; inadvertent removal of the second rib, instead of the first, should be considered a serious surgical mistake rather than a technical or expected complication of the procedure [41].

#### 6.2.2. Infraclavicular Approach

The infraclavicular approach allows an excellent view of the costo-clavicular space and could be an excellent approach for venous TOS. First described by Gol in 1968 for treatment of neurogenic TOS, the infraclavicular approach is nowadays utilized in the case of venous TOS, providing a clear view of the subclavian vein and first rib in the costo-clavicular space [42]. Among the many advantages of this approach, there is the possibility of direct visualization of the central portion of the subclavian vein, particularly when careful venolysis is required; when further medial exposure is needed, this approach can be easily shifted to trans-manubrial incision by splitting the sternal manubrium. A transverse incision—performed below the clavicle and overlying the first rib—extends from the lateral edge of the sternal manubrium to the delto-pectoral groove. The anterior aspect of the first rib is visualized and the insertion of the subclavius onto the first rib is transected. Moreover, dissecting directly on the superior border of the first rib, the insertions of the anterior and middle scalene muscles onto the first rib are divided; similarly, proceeding on the inferior aspect of the first rib, the intercostal muscles are sectioned. The pleura is then detached from the internal surface of the rib and—once the first rib is completely freed from its attachments—it is divided at the costo-manubrial junction and as close as possible to the vertebral transverse process. Complete subclavian venolysis is then required to free the vein from any residual scar tissues; if more proximal exposure for surgical veinpatch angioplasty is required, the transmanubrial approach can be easily performed by splitting the manubrium of the sternum, making medial claviculectomy unnecessary.

#### 6.2.3. Paraclavicular Approach

This approach starts with a conventional supraclavicular incision allowing exposure of the scalene fat pad.

Some minute supraclavicular cutaneous nerves that cross the operative field can be easily divided whenever it is necessary to provide better exposure. The omohyoid muscle is isolated and its middle part is resected. Appropriate isolation and lateral mobilization of the scalene fat pad is paramount during this approach. The phrenic nerve must be properly visualized on the surface of the anterior scalene muscle, and the thoracic duct has to be identified at the medial border of the scalene fat pad running toward the confluence of the internal jugular vein and the subclavian vein, particularly when operating on the left side. The insertion of the anterior scalene muscle on the Lisfranc tubercle of the first rib is sectioned under direct vision; the inferior edge is dissected from the underlying subclavian artery, brachial plexus and extrapleural fascia. The anterior scalene muscle is dissected as far as the C6 transverse process and then removed, thus making it possible to properly visualize all of the nerve roots of the brachial plexus. After retracting the brachial plexus medially, it is possible to visualize and divide the insertion of the middle scalene muscle on the posterolateral aspect of the first rib. The portion of the middle scalene muscle localizing anteriorly to the long thoracic nerve is resected and any additional part of the middle scalene muscle is freed from the posterior aspect of the first rib. As the resection of the medial part of the first rib is essential to properly treat venous TOS—considering that the subclavian vein passes below the clavicle and over the medial part of the first rib very close to the costo-clavicular junction—a further incision is required to completely remove the anteromedial part of the first rib. An additional transverse incision is therefore performed below the medial clavicle, continuing for at least 5–6 cm from the medial edge of the sternum toward the ipsilateral axilla. The cartilaginous anterior part of the first rib is then properly exposed and the subclavius muscle insertion, the costoclavicular ligament, and the muscles of the first intercostal space are detached from the first rib. The cartilaginous part of the first rib is then transected close to the sternum and the first rib is then freed from any other remaining soft tissues and completely removed [43].

#### 6.2.4. Transmanubrial Approach

**Figure 2:**
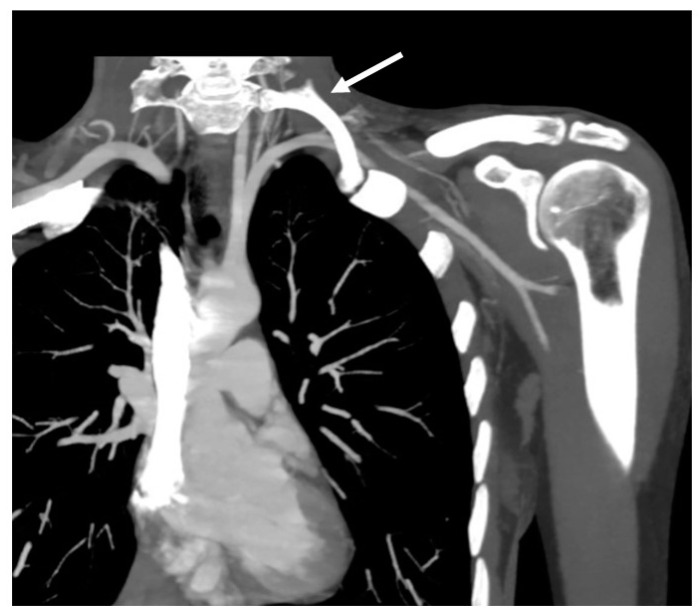
CT scan disclosing left subclavian artery compression by complete supernumerary cervical rib (white arrow).

**Figure 3:**
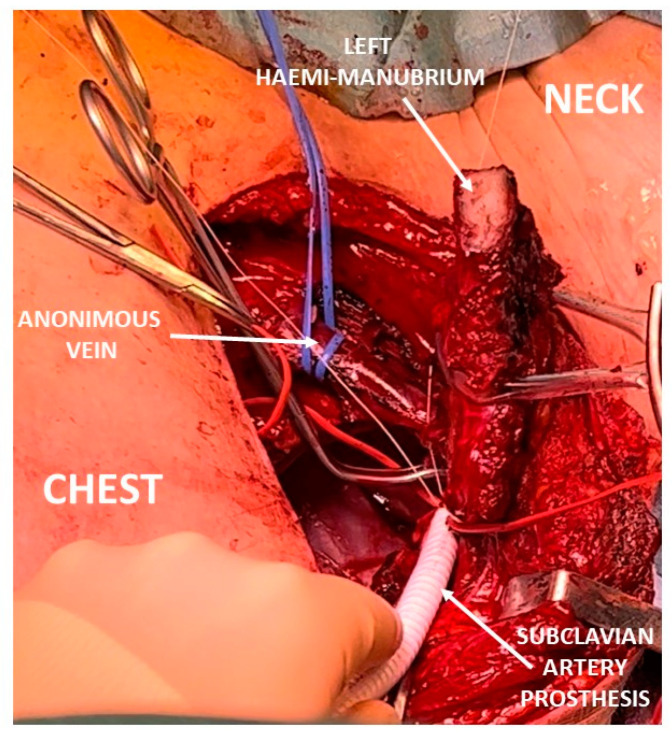
Intraoperative view of subclavian artery prosthetic reconstruction.

The transmanubrial approach was first described by Grunenwald and Spaggiari in 1997 for resecting lung cancers involving apical and anterior structures of the chest cavity, thus supplanting the transclavicular technique, which resulted in shoulder instability with functional and cosmetic consequences [44]. In the case of venous TOS requiring extensive dissection and isolation of the subclavian vein and anonymous vein with global control of the upper mediastinum vessels, the splitting of the manubrium of the sternum extending to the first intercostal space is recommended, as described by Molina in 1998 [45]. The sternal notch is properly exposed by an inverse L-shaped incision; digital dissection of the retrosternal space is performed to free the posterior aspect of the manubrium from any vascular connections. A sternal saw is used to split the sternum both vertically and horizontally (L-shaped), thus allowing a safe hemi-manubrium retraction and good exposure of the mediastinal surrounding structures. When proper isolation of the subclavian vessels is needed, a section of the costo-clavicular ligament by Gigli saw and electrocautery is accomplished, followed by anterior and middle scalene muscle disinsertion form the first rib, resulting in complete exposure from the axillary vessels to the mediastinal vessels. This approach provides excellent exposure of the subclavicular area, allowing an optimal view of both the subclavian artery and vein and a safe vascular reconstruction whenever needed. On the contrary, in the case of a supernumerary cervical rib, this technique does not offer a proper view of the take-off of the supernumerary cervical rib and the superior and middle trunk of the brachial plexus, as the vertical part of the L-shaped incision is performed anteriorly to the sternocleidomastoid muscle. For this reason, we recently proposed a modified trans-manubrial approach in which the vertical incision in the neck is performed posteriorly to the sternocleidomastoid muscle, thus properly exposing the cervical spine and brachial plexus trunks [46] (Figure 2 and Figure 3).

#### 6.2.5. Scalenotomy

**Figure 4:**
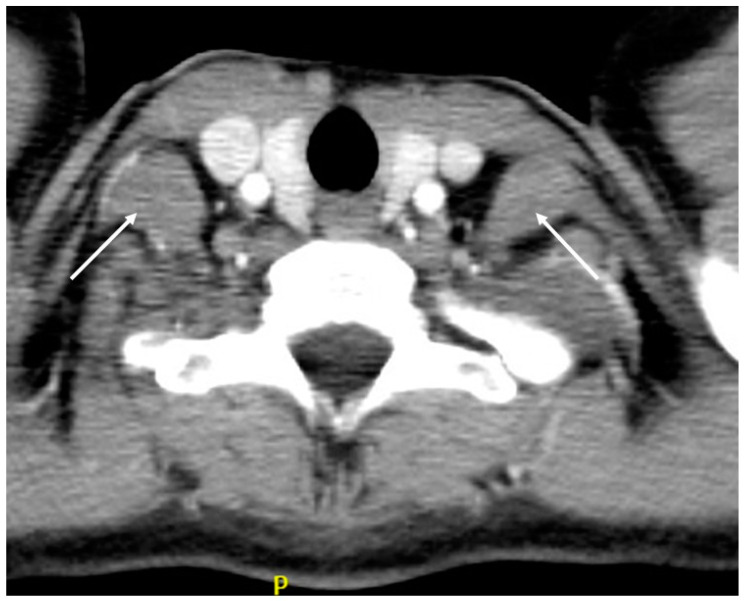
CT scan disclosing bilateral anterior scalene muscle hypertrophy (white arrows).

**Figure 5:**
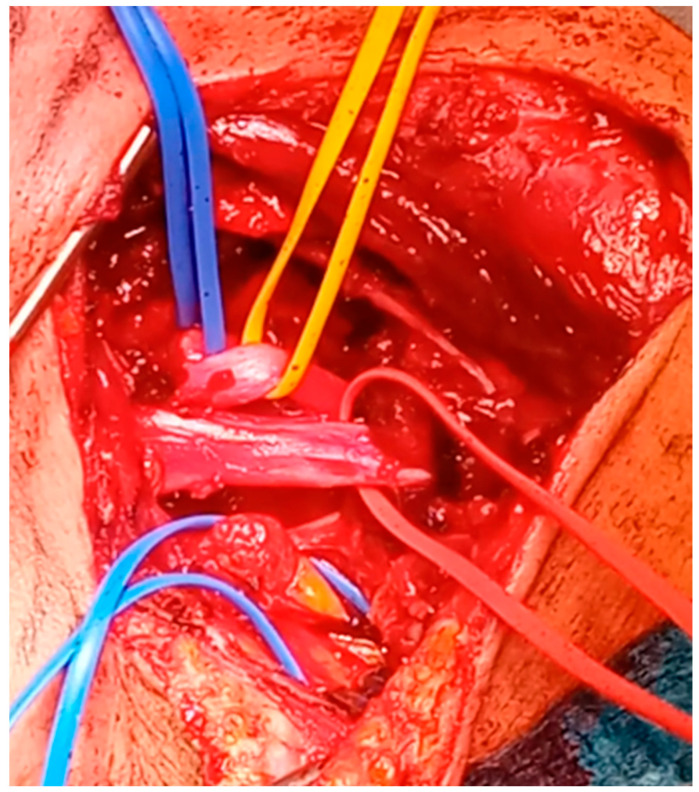
Intraoperative view of scalenotomy: upper blue vessel loop: superior trunk of the brachial plexus (C5 and C6 roots); yellow vessel loop: middle trunk of the brachial plexus (C7 root); red vessel loop: lower brachial plexus trunk (C8 and T1 roots).

Supraclavicular scalenotomy and external neurolysis without first rib resection is a surgical option, particularly in the case of post-traumatic neurogenic TOS. When patients present symptomatic improvement after local anesthetic injection in the anterior scalene muscle, they will benefit from this surgical approach. In addition, this surgical option has to be taken into consideration when clinical symptoms have not resolved after at least 3 months of physiotherapy. The surgical incision consists of a horizontal tract, parallel to the clavicle, in the supraclavicular fossa and a vertical tract, parallel to the posterior border of the sternocleidomastoid muscle. The sternocleidomastoid fibers are split and—after transection of the platysma—the scalene fat pad is isolated and then resected. This allows good visualization of the anterior scalene muscle and phrenic nerve, which is isolated and spared. The upper and middle nerve trunks of the brachial plexus are identified and dissected from the muscles. The anterior scalene muscle is then divided layer by layer until the lower nerve trunk is exposed. The middle scalene muscle is dissected and divided in a similar way and a final neurolysis of the anterior surface of the plexus is performed [47]. This approach is particularly effective in the case of scalene muscle hypertrophy [Figure 4 and Figure 5].

## 7. Conclusions

In conclusion, TOS is a complex syndrome related to the compression of nerves, arteries and/or veins located in the superior thoracic outlet. Its diagnosis is based on a clinical history and specific neurogenic or vascular symptoms, as well as on imaging techniques, such as MR, CT and US, and on specific neuro-electric tests. The therapeutic approach may vary from non-invasive to surgical techniques, according to the extent of symptoms and the patient’s condition.
